# Optimization of Cyanide-Free Composite Electrodeposition Based on π-π Interactions Preparation of Silver-Graphene Composite Coatings for Electrical Contact Materials

**DOI:** 10.3390/nano14161349

**Published:** 2024-08-15

**Authors:** Luyi Sun, Xin Chen, Ming Zhou, Jingwei Gao, Chaogui Luo, Xiao Li, Shengli You, Mingyue Wang, Gangqiang Cheng

**Affiliations:** 1 School of Mechanical and Automotive Engineering, Guangxi University of Science and Technology, Liuzhou 545006, China; 2 Guangxi Earthmoving Machinery Collaborative Innovation Center, Liuzhou 545006, China; 3 Guangxi Tsinglube New Material Technology Co., Ltd., 279 Feilu Avenue, Luzhai County, Liuzhou 545006, China; 4 Guangxi Jianxing Guangyin New Material Technology Co., Ltd., Nanning 530024, China; 5 Chengdu Carbon Co., Ltd., Chengdu 610100, China

**Keywords:** composite coating, graphene (G), π-π interaction, cyanide-free silver plating, electrical contact material

## Abstract

With the rapid development of industrial automation and power electronics, the requirements for electrical contact materials are increasing. However, traditional electrical contact materials encountered significant bottlenecks in terms of performance enhancement and production environmental friendliness. Therefore, this paper proposes a new material design idea that utilizes π-π interactions between graphene and compounds with conjugated structures in order to achieve uniform dispersion of graphene in the metal matrix and thus enhance the performance of composites. Based on this design idea, we used nicotinic acid, which has a conjugated structure and is safe, as the complexing agent, and successfully prepared high-quality silver-graphene (Ag-G) composite coatings with graphene uniformly dispersed in the metal matrix on copper substrates by composite electrodeposition technique. Subsequently, the mechanical properties of composite coatings were investigated by hardness test and X-ray diffractometer, and the tribological properties of the composite coatings and the comprehensive performance under the current carrying conditions were systematically evaluated by using friction and wear tester and load key life tester. The results show that the Ag-G composite coatings have significant advantages in mechanical, tribological, and current carrying conditions. This result not only verifies the feasibility of the design idea of the material, but also provides a new direction for the research and development of electrical contact materials.

## 1. Introduction

The performance of electrical contact materials, as a core component of relays, contactors, micro-motors, rail transportation, automotive industry, and new energy and smart grid, is directly related to the stability, reliability, and safety of electrical equipment [1,2]. With the rapid development of industrial automation, power electronics, new energy, and other fields, the requirements for electrical contact materials are getting higher and higher, and these materials must have excellent electrical conductivity, thermal conductivity, smaller contact resistance, better resistance to melt welding, and longer service life [3]. Among the electrical contact materials, silver-based electrical contact materials were most widely studied and applied due to their low resistivity and low contact resistance [4,5]. However, silver-based electrical contact materials have some problems in practical applications. First of all, high-purity silver metal has low wear resistance, melting point, and hardness, and when it is exposed to sulfur- or sulfide-containing media, its surface easily forms a silver sulfide film, which leads to susceptibility to abrasion, corrosion, and arc ablation in practical applications [6,7]. Secondly, most of the current domestic and international silver plating production lines rely on the cyanide complexation system, and although this system is a mature process, the cyanide used as a complexing agent in it poses significant environmental pollution and human health risk problems [8].

In response to the aforementioned performance drawbacks of electrical contact materials, researchers conducted a series of in-depth studies. They attempted to add second phases such as graphite, molybdenum disulfide, and nickel to some metal matrices with the aim of improving the performance of the materials. These second phases not only act as solid lubricants but also refine the grain size of the plated metal. It was shown that these second phases, while improving the tribological properties of the materials to some extent, are often accompanied by a decrease in electrical conductivity [9,10,11]. To overcome this challenge, Wang et al. [12] introduced carbon nanotubes (CNTs) with excellent electrical properties based on previous studies and synthesized CNTs-Ag-graphite electrical contact composites by powder metallurgy. This composite material utilizes the special adsorption of carbon nanotubes on graphite to promote the formation of more continuous lubrication film during the friction process. Meanwhile, thanks to the excellent electrical properties of carbon nanotubes themselves, its incorporation significantly improves the electrical conductivity of the composites. The study shows that the tribological and electrical properties of the composites are much better than those of the traditional composites with the same graphite content, but the electrical conductivity is still slightly inferior to that of the pure silver electrical contact materials.

Graphene, as an emerging material, attracted much attention from researchers for its excellent electrical [13,14], thermal [15], and mechanical [16] properties. The two-dimensional structure of graphene endows it with an extremely high specific surface area [17], which enables it to provide more contact area when interacting with the substrate material, thus effectively facilitating the transfer of electrons, phonons, and mechanical stresses. These unique properties make graphene a highly promising reinforcing phase for metal matrix composites [18]. Wang et al. [19] investigated the properties of silver coatings, silver-graphite coatings, and silver-graphene coatings on copper substrates. In silver-graphene coatings, graphene refines the metal grains through the grain boundary pinning effect on one hand and reduces the diffusion rate of electrons and ions near grain boundaries on the other hand. Studies showed that the silver-graphene coating has the best tribological properties, corrosion resistance, and the electrical conductivity is comparable to that of silver coating. In recent years, researchers found that the distribution uniformity of graphene in composites has a crucial impact on the overall performance of the material, and the uniformly distributed graphene can better produce the grain boundary pinning effect and the diffusion strengthening effect, which helps to improve the mechanical properties, electrical properties, and corrosion resistance of composites [20,21,22,23]. Therefore, the potential of graphene as a reinforcing phase in composite coatings is yet to be fully utilized, and it is expected that the performance of composite coatings will be further improved in the future by further improving the homogeneity of graphene in the metal matrix.

In the preparation of silver-based electrical contact materials, the cyanide silver plating system was widely used for its excellent complexing ability. In this system, cyanide, as a complexing agent, is able to form stable complex ions (e.g., [Ag(CN)_2_]^−^) with silver ions, thus effectively avoiding the formation of insoluble precipitates from silver ions and forming a pure and homogeneous silver coating on the surface of the workpiece through electrochemical processes [8]. However, the highly toxic nature of cyanide does pose a serious threat to the human body and the environment. In order to solve this problem, researchers actively explored and investigated a number of cyanide-free complexing agents as alternatives, among which the more studied ones include thiosulfate [24,25], butyldiimide [26], nicotinic acid [27], and so on. On this basis, some researchers prepared composite coatings by adding graphene to the cyanide-free silver coating system of succinimide. It was shown that although the mechanical properties of the composite coating were improved, there was still the problem of uneven dispersion of graphene leading to the rough surface of the coating and the degradation of electrical properties [19].

In order to overcome the challenge of graphene’s tendency to agglomerate due to its large specific surface area, which leads to uneven dispersion in the metal matrix, we propose a material design idea. Based on the existence of π-π interactions between graphene and compounds with conjugated structures [28], we envision utilizing this force to inhibit the agglomeration of graphene and thus ensure the uniform distribution of graphene in the metal matrix. Based on this material design idea, we chose nicotinic acid, which is safe and environmentally friendly and has a conjugated structure, to replace the traditional cyanide among many cyanide-free complexing agents. It can both complex silver ions and attract graphene through π-π interaction force, thus effectively preventing its agglomeration. Based on the above material design idea, we prepared Ag-G composite coatings with the cyanide-free electrodeposition method using nicotinic acid complexation, and carried out in-depth studies on their mechanical and tribological properties as well as comprehensive performance under actual current carrying conditions to verify the feasibility of this material design idea.

## 2. Materials and Methods

### 2.1. Preparation of Coating

The graphene used in this study was purchased from Shenzhen Suiheng Graphene Technology Co., Ltd. (Shenzhen, China) With the following main parameters: number of layers: 1–3 layers, sheet size: 7–12 μm, and carbon content: 98%. Silver nitrate, ammonium acetate, potassium carbonate anhydrous, potassium hydroxide and ammonia solution were purchased from Xilong Science Co., Ltd. (Shantou, China). Nicotinic acid, sodium thiosulfate, potassium disulfite, thiosemicarbazide, ethylene imine polymer, sodium dodecyl sulfate, sodium laurylsulfonate and polyethylene glycol 400 were purchased from Maikelin Science Co., Ltd. (Shanghai, China). Table 1 details the complete composition of pure Ag plating solution with nicotinic acid as the complexing agent, Ag-G composite plating solution and Ag-G composite plating solution with sodium thiosulfate as the complexing agent, as well as the operating parameters. Preparation of composite plating solution: First, 0.5 g of graphene was weighed and added to a beaker, followed by a small amount of the pure silver plating solution to ensure the initial wetting of the graphene. Then, sodium dodecyl sulfate, sodium laurylsulfonate and polyethylene glycol 400 were added sequentially in a ratio of 10 wt% each relative to the graphene concentration. The mixture was subjected to sonication equipment for 15 min to achieve uniform dispersion of the components. Subsequently, the mixture was stirred for 5 min with an electromagnetic stirrer. Afterwards, the stirred mixture was added to the sterling silver plating solution. Finally, ultrasonication of the mixed solution was continued for 30 min, resulting in 1 L of Ag-G composite plating solution.

**Figure 1:**
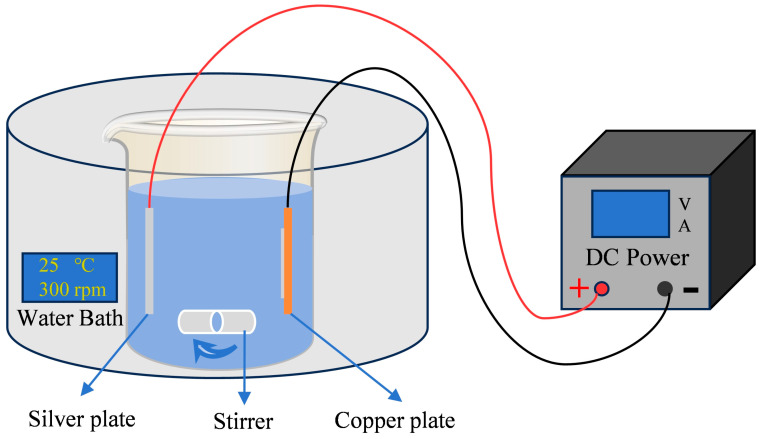
Schematic diagram of electrodeposition.

The substrate used in this study was a copper substrate with the following dimensions: 50 mm × 50 mm × 0.8 mm. The substrate was completely covered with adhesive tape, and then the excess gas was expelled, leaving an area of 30 mm × 10 mm as the silver-plated area. The substrate was cleaned with pure water and then sanded with P400, P800, and P1200 sandpaper until bright, and the surface roughness of the substrate was within the range of 0.025–0.030 μm after sanding. Then it was subjected to ultrasonic cleaning with pure water for 5 min, alkaline washing (50 g/L sodium hydroxide solution; temperature: room temperature, washing time: 90 s), and acid washing treatment (10% sulfuric acid solution, temperature: room temperature, and washing time: 90 s). Electrodeposition was carried out in a 1 L capacity beaker (as shown in Figure 1).

### 2.2. Preparation of Electrical Contacts

In order to prepare the electrical contacts, the tape covering the composite-coated substrate is first removed to ensure that the coated surface is completely bare. The coated substrate is then carefully cleaned with pure water until all residual material is completely removed. A wire cutter is used to cut out a plated area of 20 mm × 10 mm. Inside the plated area, a 5 mm diameter and 1 mm high circular table-like structure was stamped with a press and a homemade mold, which is the electrical contact. The prepared electrical contact was used for an on–off experiment.

### 2.3. Characterization and Test Methods

#### 2.3.1. Characterization Methods

Atomic force microscopy (AFM, Cypher ES, Oxford, UK) (TAP525A probe, spring constant k = 200 N/m) was used to scan and analyze the surface morphology of the coatings. A scanning electron microscope (SEM, Quanta 200FEG, OR, USA) equipped with an energy dispersive spectrometer (EDS) was used to characterize the microstructure and elemental distribution of the samples. A research-grade orthogonal materials microscope (ZEISS Axioscope 5, Oberkochen, Germany) was used to observe the surface micromorphology of the coatings. A 532 nm laser-excited Raman spectrometer (Raman, Horiba XploRA, Kyoto, Japan) was used to obtain the characteristic peaks of nanoparticles in the range of 200–3000 cm^−1^. The crystalline features of the coatings were characterized by X-ray diffractometer (XRD, Rigaku, Tokyo, Japan) in the 2θ range from 10° to 90°. The dimensions of wear spots and surface roughness were observed and analyzed using a 3D white light interferometer (BRUKER NPFLEX, Karlsruhe, Germany).

#### 2.3.2. Mechanical Property Test Methods

The hardness of the coatings was evaluated using a microhardness tester (MVC-1000Al, Shenzhen, China), where the measurement conditions were a load of 0.2 kgf and dwell time of 10 s. Ten points were measured for each sample, the maximum and minimum values were removed to ensure a small error, and the average of the remaining values was taken as the hardness.

#### 2.3.3. Tribological Performance Test Methods

The real-time coefficient of friction profiles of the coatings was tested under dry friction conditions using a friction and wear tester (BRUKER UMT-Tribolab, Karlsruhe, Germany). The system was operated under the following conditions: steel ball diameter 6.35 mm, frequency 3 Hz, stroke 3 mm, load 5 N, and duration 20 min. To minimize experimental errors, all experiments were repeated three times.

#### 2.3.4. Combined Performance Test Methods

The load key life tester (FL-8672A, Dongguan, China) was used to carry out on–off experiments to test the comprehensive performance of electrical contacts under actual current carrying conditions. The system was operated under the following conditions: a contact load of 5 N and a current carrying current of 20 A.

## 3. Results and Discussion

### 3.1. Coating Morphology

**Figure 2:**
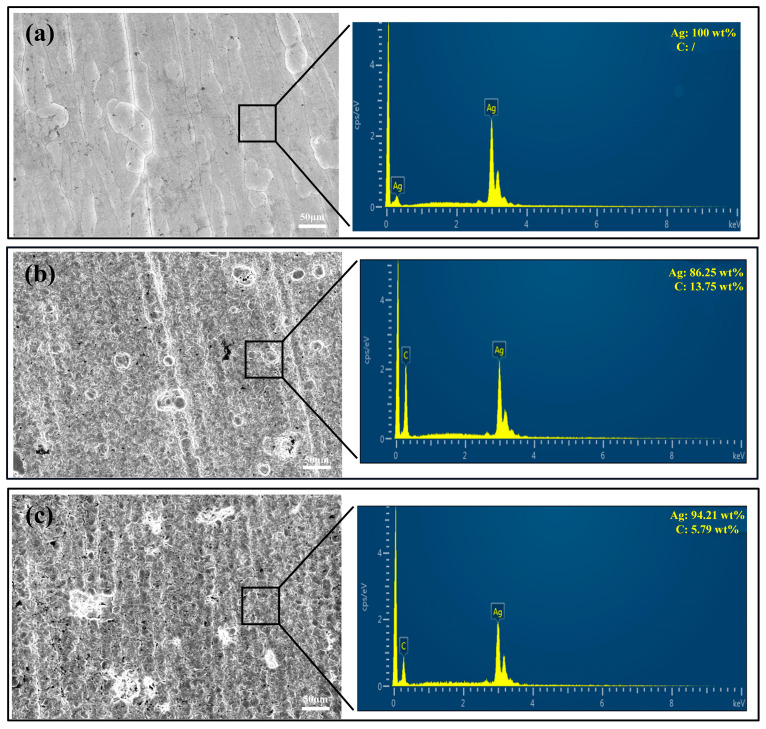
SEM images and EDS analysis of the coatings: (**a**) pure Ag coating prepared by niacin silver plating system, (**b**) Ag-G composite coating prepared by niacin silver plating system, and (**c**) Ag-G composite coating prepared by thiosulfate silver plating system.

To characterize the morphology of the coatings, two analytical tools, scanning electron microscopy (SEM) equipped with an energy dispersive spectrometer (EDS) and atomic force microscopy (AFM), were used for detailed characterization. First, we used SEM to analyze the surface of the coating. From the SEM surface morphology (Figure 2), we can observe that the surface of the pure Ag coating is extremely flat and smooth, dense, and uniform. In contrast, the Ag-G composite coating exhibits a high degree of densification, but the surface shows a large number of silver cell structures, which make the roughness of the Ag-G composite coating slightly larger than that of the pure Ag coating. This difference was mainly attributed to the graphene scales in the composite coating. Further EDS test results show that the presence of C is not detected in the pure Ag coating prepared by the silver nicotinic acid plating system, while the C content reaches 13.75 wt% on the surface of the Ag-G composite coating prepared by the silver nicotinic acid plating system, and the C content is only 5.79 wt% on the surface of the Ag-G composite coating prepared by the thiosulfate system. This shows the advantage of the nicotinic acid silver plating system.

**Figure 3:**
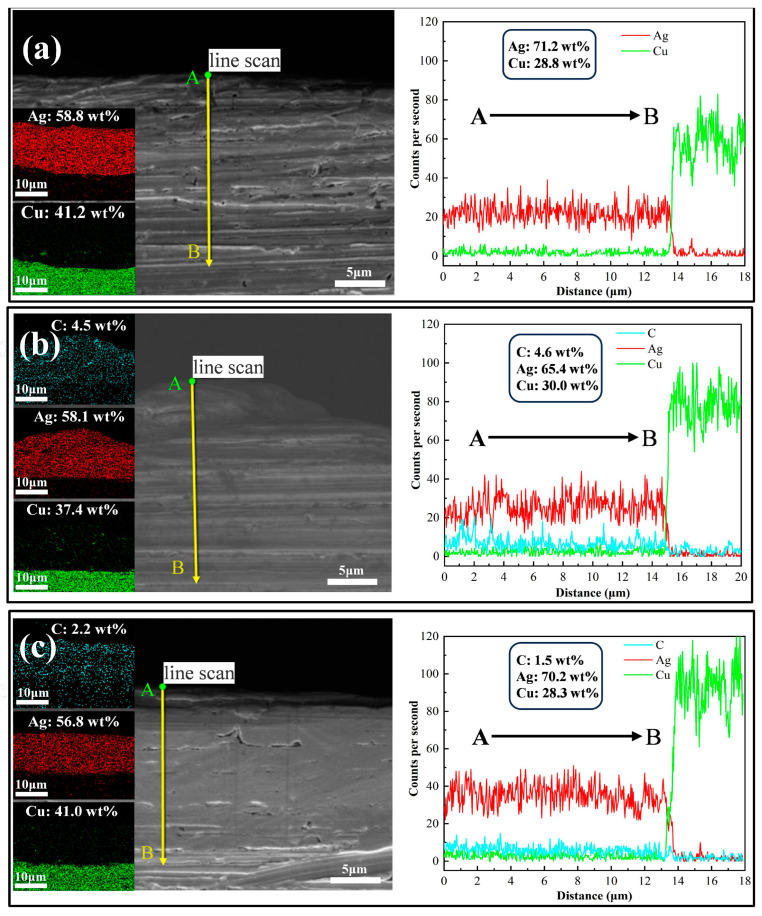
SEM images and EDS surface scan and line scan results of the coatings in cross-section: (**a**) pure Ag coating prepared by a niacin silver plating system, (**b**) Ag-G composite coating prepared by a niacin silver plating system, and (**c**) Ag-G composite coating prepared by a thiosulfate silver plating system.

Subsequently, we analyzed the cross-section of the coating using SEM and EDS. Figure 3 illustrates the SEM images and EDS surface scan and line scan results for the three coating cross-sections. Firstly, through the scanning electron microscope images, we can find that all the coatings are well bonded to the copper substrate, and no separation of the coatings from the copper substrate or obvious plating defects are observed, this result indicates that the adhesion between the substrate and the coatings is good during the plating process in this study. Then, the results of surface and line scans by EDS show that no element C was detected in the cross-section of the pure silver coating prepared by the silver nicotinic acid plating system. On the other hand, element C was detected in the cross-section of Ag-G composite coatings prepared by both the silver nicotinic acid plating system and the silver thiosulfate plating system, and the Ag-G composite coatings prepared by the silver nicotinic acid plating system were obviously higher than those prepared by the silver thiosulfate plating system. These results coincide with the EDS results of the coating surfaces, which further illustrate the advantages of the silver nicotinic acid plating system. Finally, the thickness of several coatings can be visualized by the EDS line scan results, which match our expectations.

**Figure 4:**
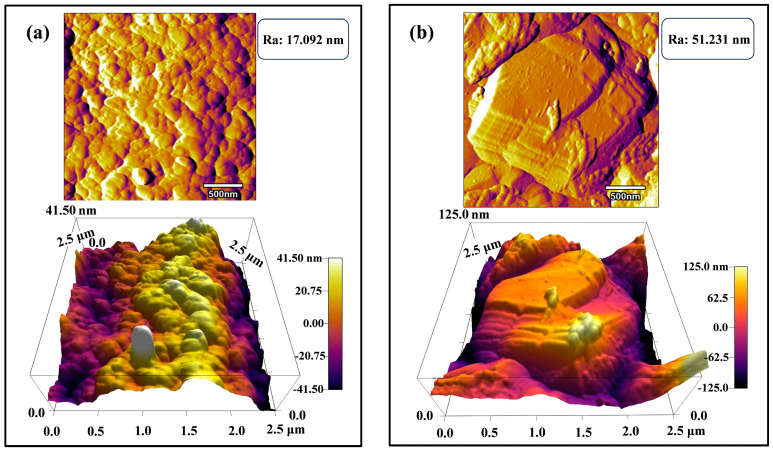
Surface morphology of (**a**) pure Ag coating and (**b**) Ag-G composite coating characterized by AFM.

Finally, we scanned the surface morphology of the coatings prepared by the silver nicotinic acid plating system using AFM. As shown in Figure 4b, compared with the surface of the pure silver coating (Figure 4a), the surface of the Ag-G composite coating has a unique graphene layered structure, which, combined with the EDS results, indicates the presence of graphene on the coating. Meanwhile, due to the presence of graphene, the localized roughness on the surface of the Ag-G composite coating (Ra = 51.231 nm) is larger than that on the surface of the pure silver coating (Ra = 17.092 nm).

### 3.2. Dispersion of Graphene on Coating

**Figure 5:**
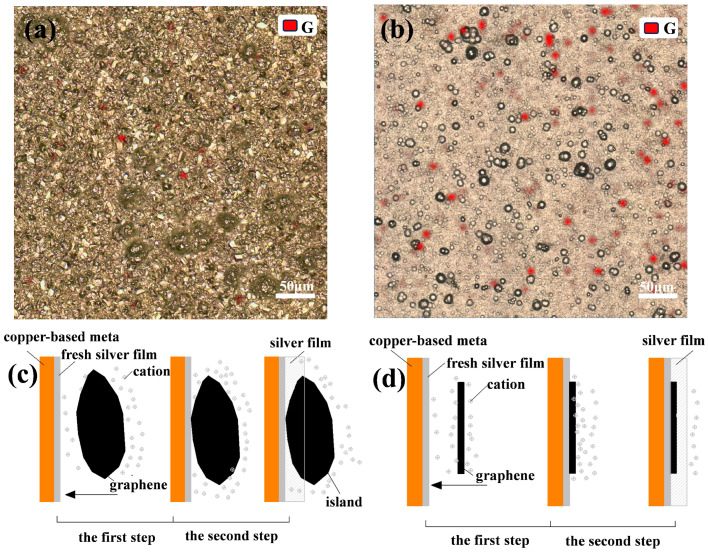
Raman surface scanning results of the coatings as well as surface morphology (**a**) Ag-G composite coating prepared by a silver thiosulfate plating system, (**b**) Ag-G composite coating prepared by a silver nicotinic acid plating system; and (**c**) schematic diagrams of the electrodeposition process under the condition of graphene agglomeration and (**d**) under ideal dispersion of graphene.

In order to investigate the dispersion of graphene in the coating, we used the Raman surface scanning technique to randomly select a 500 μm × 500 μm area on the coating and constructed a 53 × 53 matrix of points for scanning. By analyzing the Raman spectrum obtained from the scan, the most intense characteristic G peak of graphene (near 1580 cm^−1^) was selected as the target peak position [29], this results in the distribution of graphene over the scanning range, where the red part of the figure represents the presence of graphene. Figure 5a demonstrates the Raman surface scan results of Ag-G composite coatings prepared by a silver thiosulfate plating system and their surface morphology. The results show that the composite coatings prepared under this system have a low content and uneven distribution of graphene, and the surface morphology is relatively rough. On the contrary, the Ag-G composite coating prepared by the silver nicotinic acid plating system, shown in Figure 5b, has a higher content and uniform distribution of graphene, and the surface morphology is much denser and smoother. This difference in distribution is attributed to the π-π interaction between the pyridine ring of the nicotinic acid complex and graphene in the silver nicotinic acid plating system, which effectively inhibits the graphene agglomeration in the plating solution. This interaction effectively inhibits the agglomeration of graphene in the plating solution, which in turn promotes its uniform dispersion in the coating. In the silver thiosulfate plating system, there is no similar interaction between the thiosulfate complex and graphene.

Uniform distribution of graphene is essential for improving the performance of composite coatings, as it ensures that the properties of graphene can be fully utilized and form a good synergistic interaction with the Ag substrate. Figure 5c,d visualizes the formation process of the composite coating in the case of agglomeration and ideal dispersion of graphene. When graphene agglomerates, silver ions tend to be deposited on relatively uneven surfaces, which leads to an increase in overall roughness. In contrast, the surface of the Ag-G composite coating produced by the silver nicotinic acid plating system appeared smoother. The surface roughness Ra of the coatings was measured by a 3D white light interferometer, and the surface roughness of Ag-G composite coatings produced by the silver thiosulfate plating system was as high as 1.5 μm, while the surface roughness of Ag-G composite coatings produced by the silver nicotinic acid plating system was reduced to 0.30 μm, and the surface roughness of pure Ag coatings was 0.15 μm.

### 3.3. Mechanical Properties of Coating

**Figure 6:**
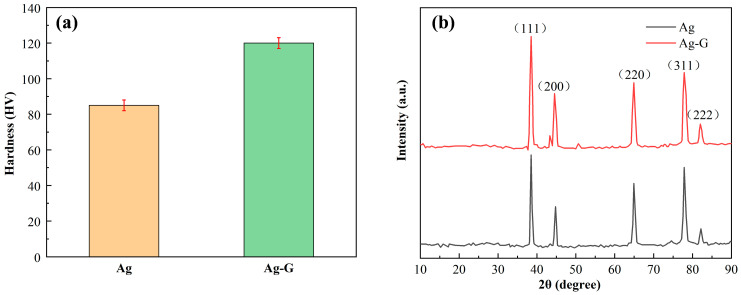
(**a**) Vickers hardness test results of pure Ag coating and Ag-G composite coating, (**b**) XRD results of pure Ag coating and Ag-G composite coating.

Hardness tests were performed on the coatings to evaluate their mechanical properties according to the above test criteria. Figure 6a demonstrates the specific results of the hardness test: the hardness value of the Ag-G composite coating is more than 120 HV, while the hardness of the pure Ag coating prepared using the same process is only about 85 HV. This comparative result clearly demonstrates the significant enhancement of the hardness of the Ag-G composite coating. According to Zhang et al. [30], the enhancement effect of nanoparticle-reinforced metal matrix composites is due to the increase in the dislocation density enhancement effect and load bearing effect. In addition, these increases can be attributed to the diffuse reinforcement as well as the difference between the matrix and particle coefficients of thermal expansion. The presence of nanoparticles not only helps to significantly refine the grains and provide more space for the formation of nuclei, but also slows down the rate of crystal growth, resulting in smaller grain size [31]. Graphene particles have superb resistance to plastic deformation. The homogeneous presence of graphene in the coating impedes the dislocation-related motion and plastic flow of grains, which ultimately produces a diffuse strengthening effect that effectively strengthens the metal coating and gives it a higher hardness [32].

The XRD results of the pure Ag coating and Ag-G composite coating are shown in Figure 6b. Comparing the Ag-G composite coatings produced by composite electrodeposition with the pure Ag coatings, we found that the main characteristic peaks did not change significantly, which indicates that the addition of graphene did not change the basic structure of the crystals themselves. To further analyze the microstructure of the coatings, we determined the corresponding half-peak widths of the pure Ag coatings and Ag-G composite coatings and calculated the grain sizes and dislocation densities by using Scherrer’s formula and its derivation, and the related results are listed in Table 2 [33,34]. In Table 2, θ represents the Bragg angle, β represents the full width of half peak (FWHM), D represents the grain size perpendicular to the lattice plane direction, and δ represents the dislocation density. By comparing the data, we find that the grain size (D) in the Ag-G composite coating is lower than that in the pure Ag coating, which indicates that the addition of graphene helps to refine the grains. In addition, the average dislocation density (δ) of the Ag-G composite coating is higher than that of the pure Ag plating, especially in the (111) plane of Ag metal. This result further demonstrates the enhancement role of graphene in composite coating, which strengthens the metal coating by refining the grains and increasing the dislocation density.

### 3.4. Tribological Properties of Coating

**Figure 7:**
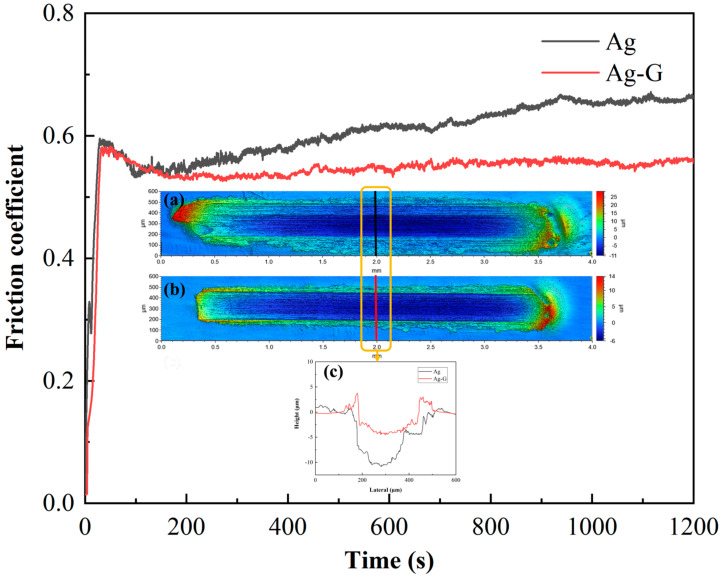
Coefficient of friction curves; (**a**) surface wear condition of pure Ag coating and (**b**) Ag-G composite coating; and (**c**) cross-section of wear scars.

In view of the above optimization effect of graphene composite electrodeposition on the coating, we initially concluded that the overall performance of Ag-G composite coating has a significant advantage over pure Ag coating. To further validate this conclusion, we conducted dry friction experiments using a friction and wear tester and performed in-depth characterization and comparison of the obtained wear data. The morphology of the wear points on the silver and composite coatings was accurately measured by white light interference 3D topography, and the cross-sections of the corresponding wear scars were plotted. As shown in Figure 7a–c, under the same experimental conditions, the wear points of the Ag-G composite coating are obviously narrower and shallower than those of the pure Ag coating. In addition, we also note that there is an obvious metal buildup at the wear edges of the Ag-G composite coating, which is mainly attributed to the high hardness of the Ag-G composite coating, and this hardness advantage effectively inhibits the edge wear of the deformed material during the friction process. In contrast, the pure Ag coating, due to the lack of an effective lubricant on the surface, was subjected to vertical and horizontal forces when in contact with the steel ball, resulting in the silver metal in the coating continuously peeling off under load and continuous friction to form wear debris, the accumulation of which further exacerbated the friction and wear effects.

Figure 7 illustrates the corresponding friction curves of the two coatings. The comparison shows that the friction coefficient of the Ag-G composite coating (μ_1_ = 0.5424) is significantly lower than that of the pure Ag coating (μ_2_ = 0.6031). This result directly reflects the superiority of the Ag-G composite coating in terms of friction performance and verifies the positive effect of graphene as a lubricant in reducing friction and improving wear resistance. For Ag-G composite coating, graphene in the coating not only acts as a reinforcing agent to improve the hardness of the coating, but also plays the role of a lubricant [35]. Under the action of friction, some of the graphene particles may be detached from the coating and uniformly distributed on the friction contact surface, forming a lubricant coating based on graphene particles. This self-lubricating property helps to reduce the direct contact of the friction interface, thus reducing the coefficient of friction and wear rate, and effectively reducing the reoccurrence of wear [36].

### 3.5. Overall Properties of the Coating

**Figure 8:**
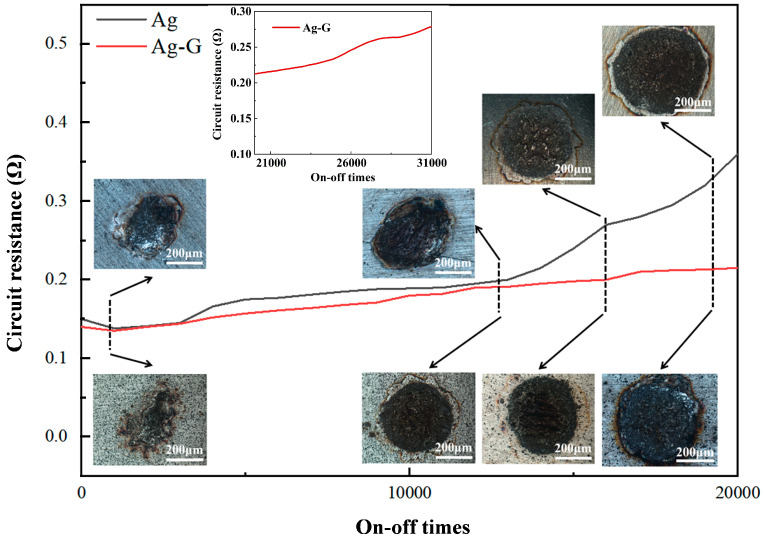
On–off experimental resistance curves and contact surface morphology of pure Ag contact and Ag-G composite contact during the on–off cycle.

The on–off experiment was conducted on the load key life tester to accurately simulate the working condition of electrical contacts under actual current carrying conditions. Compared to the dry friction experiment, the on–off experiment is more demanding because it involves not only normal wear and tear, but also the effects of high temperature arc erosion. Figure 8 shows a comparison of the resistance curves of the two electrical contacts. Due to the excellent conductivity of graphene itself, the circuit resistance of the Ag-G composite contact remained below that of the pure silver contact throughout the experiment. The on–off experiment showed a slight decrease in resistance between the 1000 to 2000 cycles. This change is mainly attributed to the increase in contact area at the microscopic level between the cathode and anode contacts at the beginning of the experiment under the effect of interfacial extrusion. At this time, the effect of arc ablation is relatively weak and almost negligible, so the resistance shows a decreasing trend instead. The situation changes during the 2000 to 13,000 cycles of the on–off experiment. As the contact surface is subjected to the combined effect of continuous mechanical impact and arc ablation, the actual contact area begins to decrease and the temperature near the contact point rises. The increase in temperature exacerbated the oxidation process of pure silver, leading to a gradual increase in contact resistance [37]. When the on–off experiment was carried out for about 13,000 cycles, a sharp increase in the circuit resistance of the pure silver contacts was observed. This change indicates that electrical ablation had a serious effect on frictional wear, probably due to a drastic change in the interfacial state as a result of severe ablation of the Ag coating or the occurrence of fusion welding on the surface.

By comparing the contact pictures in Figure 8, we clearly observe that the Ag-G composite contact is significantly lower than the pure silver contact in terms of the size of the ablation area and the degree of ablation. This intuitive comparison further verifies the significant advantage of Ag-G composite contacts over pure silver contacts in terms of ablation resistance. In this experiment, we clearly defined the criteria for the determination of contact failure: when the circuit resistance doubles, the contact is judged to have failed. Based on this criterion, we calculated the lifetime of the pure silver contacts to be in the range of approximately 19,100 to 19,300 cycles, while the Ag-G contacts have a significantly longer lifetime time, reaching a range of approximately 31,000 to 31,200 cycles. This comparison not only highlights the superior durability of Ag-G composite contacts, but also provides strong support for their widespread use in electrical contacts.

**Figure 9:**
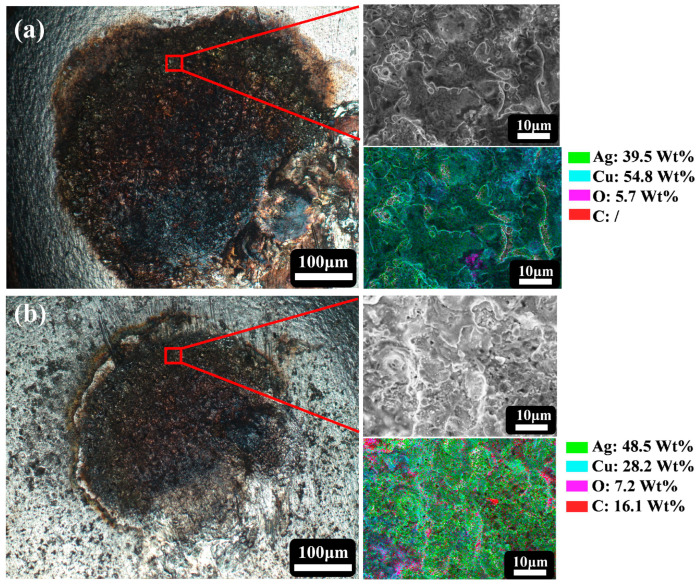
Images of surface morphology and distribution of various elements in the localized ablation region of (**a**) pure Ag contact and (**b**) Ag-G composite contact after 10,000 times of the on–off experiment.

Figure 9 demonstrates the surface morphology and elemental distribution results of pure Ag and Ag-G composite contacts after 10,000 times of on–off experiment. It is observed that copper and silver serve as the basic components of the sample, while carbon originates from the added graphene. Under the influence of arc ablation, the material clearly flows towards the peripheral edges, a phenomenon that mainly originates from the combined effect of arc action and mechanical loading. It is noteworthy that the Ag-G composite contact is significantly smaller than the pure Ag contact in terms of arc ablation area and extent, which fully demonstrates the excellent performance of the composite coating in resisting arc ablation. Compared with pure Ag contacts, Ag-G composite contacts show stronger resistance to arc ablation and grain displacement by virtue of their higher hardness. This property effectively prevents material displacement to the outer edge during the on–off experiment, thus ensuring the stability and durability of the contact.

EDS analysis shows that the percentage of silver mass in the ablation area of a pure Ag contact is only 39.5 wt%, whereas for an Ag-G composite contact it is as high as 48.5 wt%. This means that the composite contact retains more silver under the same operating conditions, ensuring that the desirable properties of the silver metal are fully retained. In practice, silver is still the key metal that dominates performance. Therefore, the higher retention of silver in composite contacts has a positive impact on the overall performance of the contacts. In addition, when two solid surfaces are in contact, the actual contact area is only a very small part of the apparent area. When the contact area is smaller, the local resistance increases. This in turn exacerbates the exotherm, leading to some degree of ablation and further deterioration of the resistance. As shown in Table 3, after 10,000 times of the on–off experiment, the roughness Ra of the Ag-G composite contacts at both cathode and anode positions remained relatively low. This is attributed to the role of graphene in the Ag-G composite coating, which effectively reduces the accumulation of localized heat and the deterioration of resistance during the on–off process.

**Figure 10:**
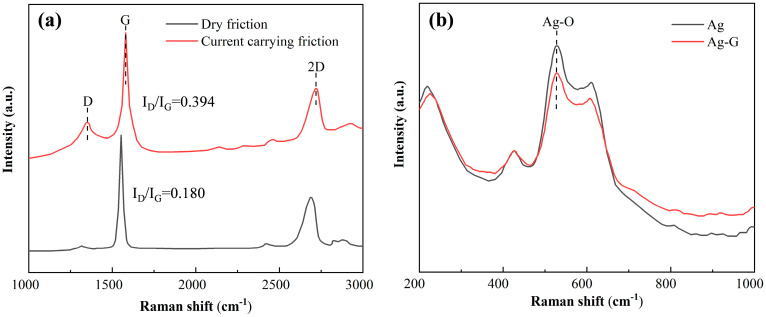
(**a**) Raman spectra of graphene residues at each wear point after different experiments. (**b**) Raman spectra of the respective ablation zones of pure Ag coatings and Ag-G composite coatings after 10,000 times of the on–off experiment.

The results are shown in Figure 10a, where we analyzed and compared the Raman spectra of graphene residues at each wear point after dry friction experiments and after 10,000 times of on–off experiment. The Raman spectra of the ablation region after 10,000 times of on–off experiment clearly show that there is a significant increase in the intensity of the D peak of graphene. In Raman spectroscopy, the D peak usually indicates the presence of defects or edges in carbon-based materials, while the G peak is often used to measure the way carbon atoms are stacked between layers in carbon-based materials [28]. The intensity ratio of the D peak to the G peak (I_D_/I_G_) is an important parameter for assessing the defect density of carbon-based materials [38,39]. According to our analysis, the I_D_/I_G_ ratio is 0.394 for the current carrying friction condition, while the I_D_/I_G_ ratio is 0.180 for the dry friction condition. These results clearly point out that the current carrying friction condition produces larger defects on graphene. This means that current carrying friction is much more destructive to electrical contacts and graphene on electrical contacts compared to dry friction. It also suggests that these graphene substitutes for silver resisted some of the damage.

Figure 10b shows the Raman spectra of the contact ablation region of pure Ag contacts and Ag-G composite contacts after 10,000 times of the on–off experiment. The peak near 500 cm^−1^ represents the Ag-O symmetric stretching vibration of the monovalent silver unit with symmetric stretching vibration, and it is obvious that the intensity of the silver oxide peak of the Ag-G composite contact is lower than that of the pure Ag contact. This result suggests that an oxide film was generated on the surface of both contacts to protect the plated metal during the on–off experiment. However, it is noteworthy that graphene on Ag-G composite contacts played a key role in this process. Graphene not only acts as a protective layer on the contact surface to reduce the occurrence of oxidation reaction, but also acts as a lubricant to reduce friction and wear, thus effectively inhibiting the oxidation process [40,41]. This inhibition ensures the material stability of Ag-G composite contacts during current breakage.

### 3.6. Composite Electrodeposition Mechanism

**Figure 11:**
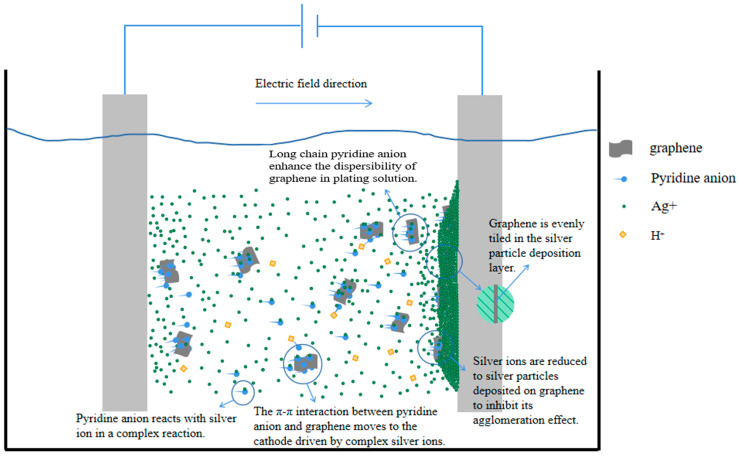
Mechanism of composite electrodeposition under niacin cyanide-free silver plating system.

The complex of the plating solution is chosen to be nicotinic acid, an organic acid containing a pyridine ring. Although nicotinic acid is slightly soluble in water, it exhibits an extremely high degree of dissociation in water. When incorporated into water, nicotinic acid molecules dissociate into ions at specific concentrations. Specifically, in the presence of negative electrode potentials and chemisorption of N atoms, special complexes are formed with the structural formula 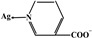. According to the relevant literature, there is a π-π interaction force between graphene with a six-membered ring structure and compounds with a conjugated structure [28], and the pyridine ring of nicotinic acid, the plating solution complex mentioned above, has a conjugated structure. Therefore, the complex is adsorbed on the graphene surface through the π-π interaction between the pyridine ring structure and graphene. On the one hand, the long chain structure of the pyridine anion inhibits the agglomeration of graphene, and on the other hand, under the action of an external electric field, the complex and the graphene as a whole move together toward the cathode and are deposited. As shown in Figure 11, these two reasons ensure the uniform distribution of graphene in the Ag-G composite coating.

## 4. Conclusions

In this study, based on the π-π interactions between graphene and compounds with conjugated structures, high-quality Ag-G composite coatings with graphene uniformly distributed in the metal matrix were successfully prepared on copper substrates using the composite electrodeposition technique. The experimental results show that this uniformly distributed graphene significantly improves the microstructure of the composite coating, resulting in grain refinement and increased dislocation density of the coating, which greatly enhances the mechanical and tribological properties of the coating, as well as the comprehensive performance under current carrying conditions.

Specifically, when the graphene concentration in the composite plating solution was 0.5 g/L, the average Vickers hardness of the prepared Ag-G composite coatings was 41% higher than that of the pure silver coatings, which exhibited excellent mechanical properties. In terms of tribological properties, the dry friction marks of the Ag-G composite coatings were significantly reduced and the average coefficient of friction decreased by 10% compared with that of the pure Ag coatings, showing lower friction loss. In the on–off experiment under current carrying condition, the Ag-G composite electrical contact shows lower surface oxidation and less edge material buildup at the wear point. These advantages make the service life of the Ag-G composite contact extend more than 62% compared with the pure silver contact, which fully proves the superiority of Ag-G composite coating in practical applications. This achievement not only verifies the feasibility of the design idea of utilizing the π-π interactions between graphene and compounds with conjugated structures to realize the uniform dispersion of graphene in the metal matrix, but also provides new ideas and methods for the future development of high-performance electrical contact materials.

## 5. Patents

Zhou M. A graphene-nicotinic acid silver plating solution and preparation method: China, 2021106681251 [P]. 2 November 2021.

## Figures and Tables

**Table 1:** Plating solution composition and deposition conditions for different coatings.

Composition and Conditions	Ag Plating Solution (Nicotinic Acid)	Ag-G Composite Plating Solution (Nicotinic Acid)	Ag-G Composite Plating Solution (Sodium Thiosulfate)
AgNO_3_ (Silver nitrate)	45 g/L	45 g/L	45 g/L
C_6_H_5_NO_2_ (Nicotinic acid)	100 g/L	100 g/L	/
CH_3_COONH_4_ (Ammonium acetate)	77 g/L	77 g/L	/
K_2_CO_3_ (Potassium carbonate anhydrous)	70 g/L	70 g/L	/
KOH (Potassium hydroxide)	45 g/L	45 g/L	/
NH_3_·H_2_O (Ammonia solution)	32 mL/L	32 mL/L	/
Na_2_S_2_O_3_ (Sodium thiosulfate)	/	/	250 g/L
K_2_S_2_O_5_ (Potassium disulfite)	/	/	45 g/L
CH_5_N_3_S (Thiosemicarbazide)	/	/	0.6 g/L
(CH_2_CH_2_NH)_n_ (Ethylene imine polymer)	/	/	0.6 g/L
C_12_H_25_SO_4_Na (Sodium dodecyl sulfate)	/	0.05 g/L	0.05 g/L
C_12_H_25_SO_3_Na (Sodium laurylsulfonate)	/	0.05 g/L	0.05 g/L
HO(CH_2_CH_2_O)_n_H (Polyethylene glycol 400)	/	0.05 g/L	0.05 g/L
Graphene	/	0.5 g/L	0.5 g/L
Temperature	25 ± 1 °C	25 ± 1 °C	25 ± 1 °C
pH	9.5–10.0	9.5–10.0	6.0–7.0
Current density	0.24 A/dm^2^	0.24 A/dm^2^	0.24 A/dm^2^
Electrolyte agitation	300 rpm	300 rpm	300 rpm
Deposition thickness	13–16 μm	13–16 μm	13–16 μm
Anode	Ag	Ag	Ag

**Table 2:** Calculated results of applying Scheller’s formula and its derivation to the XRD images of pure Ag coating and Ag-G composite coating.

Crystal Face	2θ (°)	β (°)	D (nm)	δ × 10^−3^ (nm^−2^)	Coating
(111)	38.50529	0.44091	18.87275	2.80756	Ag
(200)	44.72020	0.47910	17.73013	3.18109	Ag
(220)	64.85535	0.56278	16.53768	3.65637	Ag
(311)	77.75365	0.66403	15.19660	4.33018	Ag
(111)	38.38691	0.54018	15.39892	4.21715	Ag-G
(200)	44.55782	0.53871	15.75906	4.02660	Ag-G
(220)	64.80095	0.59909	15.53067	4.14590	Ag-G
(311)	77.71745	0.68990	14.62303	4.67654	Ag-G

**Table 3:** Results of surface roughness Ra of contact material after 10,000 on–off experiments.

Coating Layer	Pure Ag Coating		Ag-G Composite Coating	
Roughness degree	Cathode	Anode	Cathode	Anode
(Unit: μm)	3.583	4.525	3.182	3.491

## Data Availability

The data presented in this study are available upon reasonable request from the corresponding author.
